# Yoga as Adjunct Therapy for Chronic Heart Failure: A Systematic Review and Meta-Analysis of Randomized Controlled Trials

**DOI:** 10.1055/s-0043-1774738

**Published:** 2023-09-22

**Authors:** Abhijit Dutta, Aruchunan Mooventhan, L. Nivethitha

**Affiliations:** 1 International Cooperation Section, Ministry of Ayush, Government of India, New Delhi, India; 2 Department of Research, Government Yoga and Naturopathy Medical College, Chennai, Tamil Nadu, India; 3 Department of Naturopathy, Government Yoga and Naturopathy Medical College, Chennai, Tamil Nadu, India

**Keywords:** yoga, meditation, chronic heart failure, randomized controlled trials, quality of life, peak VO _2_, NT-proBNP, systematic review, meta-analysis

## Abstract

**Background** Chronic heart failure (CHF) is a prevalent cardiovascular condition that can significantly impact the quality of life and increase mortality risk. Yoga is a mind–body therapy that has been studied as a potential complementary treatment for CHF. However, the effectiveness of yoga in improving outcomes in patients with CHF remains uncertain.

**Methods** We conducted a systematic review of randomized controlled trials (RCTs) evaluating the effects of yoga on outcomes in patients with CHF. We searched the PubMed, Embase, Scopus, Cochrane Library, and IndMED databases from inception to March 2023. The outcomes of interest were left ventricular ejection fraction (LVEF), cardiac biomarkers, exercise capacity, quality of life, and cardiac function.

**Results** We identified 11 RCTs that met our inclusion criteria, involving a total of 552 participants. The meta-analysis showed that yoga was associated with significant improvements in peak VO _2_ (mean difference [MD]= 3.29; 95% Confidence Interval [CI]: 1.64 to 4.94; I ^2^ = 0%), exercise capacity (MD=101.54; 95% CI: 6.24 to 196.83; I ^2^ = 96%), quality of life (MD = –19.99; 95% CI: –25.76 to –14.22; *I* ^2^ = 43%), NT-proBNP (MD = –288.78; 95% CI: –492.20 to –85.37; *I* ^2^ = 94%), and 6-minute walk test (MD = 101.54; 95% CI: 6.24–196.83; *I* ^2^ = 96%), but not in the left ventricular ejection fraction (MD = 4.28; 95% CI: –1.14 to 9.70; *I* ^2^ = 93%). Subgroup analysis suggested that the effect of yoga on the quality of life is more pronounced in patients with the “New York Heart Association” (NYHA) class I and II CHF patients and in those who practiced yoga for longer durations. No serious adverse events related to yoga were reported. Most of the included studies were of “low” quality.

**Conclusion** Current evidence suggests that yoga may be an effective complementary and integrative therapy for improving peak VO _2_ exercise capacity, NT-proBNP, and quality of life in patients with CHF. However, the low-quality evidence does not render us to conclude anything beyond doubt or draw any firm clinical recommendation. Future high-quality studies are needed to explore the optimal duration and frequency of yoga practice and its effects on long-term outcomes in this population.

## Introduction

According to the American Heart Association/American College of Cardiology guidelines, heart failure (HF) is defined as “a complex clinical syndrome that can result from any structural or functional cardiac disorder that impairs the ability of the ventricle to fill or eject blood.” 1 HF is a global epidemic that affects an estimated 23 million people and the leading cause of substantial numbers of morbidity, hospitalizations, mortality, and health care costs, worldwide. 2 3 Infectious illnesses and/or dietary deficiencies are no longer the primary causes of death and morbidity in Asian nations, but rather diseases linked to a sedentary lifestyle, such as cardiovascular disease (CVD). 2 According to disease-specific estimates of HF prevalence and incidence rates, the prevalence of HF in India ranges from 1.3 to 4.6 million, with an annual incidence of 491,600 to 1.8 million due to coronary artery diseases, hypertension, obesity, diabetes, and rheumatic heart diseases. 4 Reduced physical function, increased dyspnea, and weariness are all signs of HF. The quality of life (QoL) is also reduced in HF patients. 5 Despite recent advancements in pharmacologic and device treatment, cardiovascular morbidity and death remain high. 6 Likewise, consistent use of conventional medication can lead to various adverse effects. Hence, there is a need for an alternative, nonpharmacologic approach like yoga that may improve physical and psychological function. 5

Yoga is a mind–body practice that incorporates physical postures, breathing methods, and meditation to promote relaxation, stress reduction, and general health and well-being. It grew and evolved as a dynamic way of life and spiritual practice in India. 4 While the benefits of yoga have been studied in a variety of populations and conditions, its effects on chronic HF remain unclear. However, yoga has gained immense popularity and is considered beneficial in cardiac rehabilitation. 7 8 HF is associated with altered autonomic function, resulting in markedly elevated sympathetic activity and blood pressure. 4 Yoga has recently gained popularity as a practice in Western culture. Yoga includes breathing exercises, relaxation techniques, and meditation in addition to physical activities. By increasing baroreflex sensitivity and heart rate variability (HRV), lower breathing rate can boost vagal activation and lessen the effect of the sympathetic branch of the autonomous nervous system. Blood pressure and heart rate may drop as vagal involvement increases. Increased systolic stroke volume and enhanced left ventricular ejection fraction (LVEF) may result from load reduction. 9

Various systematic reviews are available on yoga for CVD risk factors, 10 heart disease in general, 11 secondary prevention, 12 and hypertension. 13 14 15 Although an increasing number of studies have been published in the past few years, there is only one systematic review and meta-analysis 16 performed to evaluate the effect of yoga in patients with HF. The review, published in 2014, included two randomized controlled trials (RCTs) and primarily measured peak VO _2_ and health-related QoL (HRQoL). It indicates a lack of updated systematic review and meta-analysis in yoga and HF. Moreover, several trials have been published since the review was conducted in yoga and CVDs, especially HF.

Given the potential promise of yoga as a complementary and integrative therapy for chronic heart failure (CHF), there is a need for a comprehensive and up-to-date systematic review and meta-analysis of the available evidence. This review aims to synthesize the findings of RCTs that have investigated the effects of yoga on biomarkers and QoL in patients with CHF. By examining the collective evidence, we hope to provide insights into the potential benefits and limitations of yoga as an adjunct therapy for this challenging health condition.

## Methods

### Research Question

What is the effect of yoga on cardiac biomarkers, function, and QoL in individuals with CHF?

### Transparency

This review was performed in accordance with Preferred Reporting Items for Systematic Reviews and Meta-Analyses (PRISMA) guidelines.

### Eligibility Criteria

**Types of studies:** RCTs that evaluated the effect of any type of yoga in patients with CHF (systolic and/or diastolic) compared with either standard medical care or other similar interventions, published in English, were included. Furthermore, crossover or non-RCTs, single-group pre–post trials, observational studies, case series, case reports, review articles, surveys, and health news were excluded.

**Types of participants:** Studies enrolled patients with systolic and/or diastolic HF (aged >18 years) were included in this review. To be eligible, a trial required patients with HF to be randomized to at least one group receiving any type of yoga intervention. CHF is defined as a clinical syndrome characterized by symptoms and/or signs of HF with objective evidence of structural or functional cardiac abnormalities. Yoga intervention could be defined as any practice that involves physical postures (asanas), breathing techniques (pranayamas), meditation (dhyana), and/or relaxation exercises.

**Types of outcome measures:** The effects of yoga could be evaluated through outcomes like cardiac biomarkers, function, and QoL. Therefore, considering the context, the main outcomes of interest were as follows:

- *LVEF* : It is a measurement of the amount of blood that is pumped out of the left ventricle of the heart with each heartbeat. It is expressed as a percentage and is calculated by dividing the volume of blood pumped out of the left ventricle during systole (the contraction phase) by the total blood volume in the left ventricle at the end diastole (the relaxation phase).
- *N-terminal pro-brain natriuretic peptide (NT-proBNP)* : It is a biomarker commonly used in clinical practice to aid in diagnosing and managing HF. NT-proBNP is a cleavage product of the pro-brain natriuretic peptide (proBNP), which is synthesized and secreted primarily by the heart ventricles in response to increased pressure or volume.
- *
Peak VO _2_* : It measures an individual's maximum oxygen consumption during exercise and is commonly used to assess cardiorespiratory fitness. An improvement in peak VO _2_ suggests an improvement in cardiorespiratory fitness, which can have positive health implications.
- *Other outcomes related to CHF* : exercise capacity, 6-minute walk test (6mWT), hemodynamic changes (heart rate and systolic and diastolic blood pressures), inflammatory markers, hormone levels, QoL, etc.

In addition, we excluded mental health outcomes, which were not the objective of interest of this study.

### Search Methods for Identification of Studies

A rigorous and systematic search strategy was formulated to identify potential articles investigating the impact of yoga on CHF. The search was conducted across multiple electronic databases, including PubMed, Embase, Scopus, Cochrane Library, and IndMED, utilizing appropriate keywords and MeSH terms related to yoga and CHF. The search was not restricted by language; only studies published until March 31, 2023, were included. In addition to the electronic database search, a snowballing technique was utilized to identify other relevant studies by searching through the bibliographies of important articles. The exclusion criteria were appropriately applied to exclude book chapters, abstracts, incomplete reports, case reports, and duplicate records. By adopting such a comprehensive and rigorous approach, only relevant and high-quality studies were considered for inclusion in the synthesis. The search strategy used for PubMed is described in Table 1 , which is modified and adapted as per the suitability of other databases.

**Table 1:** Strategy for PubMed search

Sl no.	Search terms
1	“Diastolic heart failure” [Title/Abstract] OR “Diastolic dysfunction” [Title/Abstract] OR “Systolic heart failure” [Title/Abstract] OR “Ejection Fraction” [Title/Abstract] OR “Heart Failure” [Mesh] OR “Heart Failure” [Title/Abstract] OR “Cardiac Failure” [Title/Abstract] OR “Heart Decompensation” [Title/Abstract] OR “Pulmonary Heart Disease” [Mesh] OR “Pulmonary Heart Disease” [Title/Abstract] OR “Cor Pulmonale” [Title/Abstract]
2	Meditation [Title/Abstract] OR “Relaxation Technique” [Title/Abstract] OR “Breathing Exercise” [Title/Abstract] OR “Nostril Breathing” [Title/Abstract] OR Pranayam* [Title/Abstract] OR “Yoga” [Mesh] OR “Yoga” [Title/Abstract] OR “Yogic” [Title/Abstract] OR “Asana” [Title/Abstract] OR “Pranayama” [Title/Abstract] OR “Dhyana” [Title/Abstract]
3	randomized controlled trials as topic [MeSH terms] OR “Randomized Controlled Trial” [Title/Abstract] OR “Random Allocation” [Title/Abstract] OR “Double Blind” [Title/Abstract] OR “Single Blind” [Title/Abstract] OR “Clinical trial” [Title/Abstract] OR “Control” [Title/Abstract]

### Data Collection and Analysis

**Assessment of study eligibility** : The identified studies were screened for relevance and eligibility by two independent reviewers, who assessed the titles and abstracts for the inclusion criteria based on the research question. Full-text articles were obtained for studies that met the inclusion criteria or whose relevance was unclear based on the abstract. The eligibility of the full-text articles was also assessed independently by two reviewers, and any disagreements were resolved through discussion or with the involvement of a third reviewer.

**Data extraction:** The relevant data from the eligible studies, such as study characteristics (e.g., author, year, country, sample size), participant characteristics (e.g., age, sex, diagnosis), intervention characteristics (e.g., type, frequency, duration), outcome measures (e.g., biomarkers of HF, QoL), and study results, were independently extracted by two reviewers. Any discrepancies that arose during the data extraction process were resolved through discussion or consultation with a third reviewer. In case of incomplete data reporting in the eligible studies, the corresponding authors were contacted for additional information. The data were considered incomplete if the authors did not respond after successive two reminders. This approach ensures that robust efforts were made to obtain complete data and that the analysis is based on the most comprehensive and accurate information available.

**Quality assessment of included studies:** To assess the risk of bias (RoB) in individual studies, two reviewers independently utilized the Cochrane collaboration tool for assessing RoB, Version-2 (RoB 2.0). 17 The RoB was evaluated across six domains, which include selection bias, performance bias, detection bias, attrition bias, reporting bias, and other biases.

“Selection bias” refers to the way participants are chosen for a study. This review used two criteria to assess selection bias: “random sequence generation” and “allocation concealment.” “Adequate random sequence generation” means that participants are placed into groups randomly, and “adequate allocation concealment” means that the researchers could not predict which group a participant would be placed in before or during the study. “Performance bias” refers to whether the participants and researchers are aware of which group a participant is in. “Adequate blinding” means that neither the participants nor researchers knew which group the participant was in. Although it may be challenging to blind participants and researchers in yoga trials, it is still essential to minimize bias. “Detection bias” refers to whether the outcome assessors know which group a participant is in. Adequate blinding of the outcome assessors is essential to prevent bias. “Attrition bias” occurs when some participants drop out of the study, leading to incomplete data. If more than 20% of participants drop out, it can affect the study results, so addressing this with an intention-to-treat analysis is crucial. “Reporting bias” occurs when not all the study's outcomes are reported. “Other sources of bias” that do not fit into any of these categories are also possible. The RoB was assessed as “low,” “unclear,” or “high” based on how well these criteria were met. Any disagreements were resolved through discussion among the reviewers.

Any discrepancies that arose during the evaluation of RoB were resolved through discussion or consultation with a third reviewer.

**Data synthesis and analysis:** We first conducted a qualitative synthesis (systematic review) of the included studies. We identified similar and extractable quantitative outcomes across studies and pooled them for quantitative synthesis (meta-analysis). For continuous outcomes (e.g., biomarkers of HF, QoL), we reported the mean difference (MD) with 95% confidence intervals (CIs). The statistical heterogeneities were measured using the *I* ^2^ statistic and Cochran's *Q* test. We used fixed effect and random effect models to estimate the average effect, depending on the amount of heterogeneity exhibited for each outcome.

However, some studies reported the difference from the baseline, rather than the final value score. We pooled the values using the mean difference (unstandardized) method in such cases. Using the mean difference method, we combined studies with change-from-baseline outcomes and studies with final measurement outcomes in a meta-analysis. This is because the mean difference method calculates the difference between two means (e.g., the mean value in the intervention group minus the mean value in the control group) and therefore does not require the outcomes to be standardized. The statistical analyses for this systematic review and meta-analysis were conducted using the “Meta” package in the R software environment ( https://cran.r-project.org/ ). Specifically, the functions “metacont” and “forest” were utilized to perform the meta-analyses and generate the forest plots, respectively.

**Subgroup and sensitivity analysis:** In the cases where there was substantial heterogeneity between studies, we conducted subgroup analyses based on participant characteristics (e.g., disease severity), intervention characteristics (e.g., type, frequency, duration), and RoB. This approach helps us to identify potential sources of heterogeneity and provides insights into the factors that may influence the effect of the intervention.

We also conducted sensitivity analyses to explore the impact of individual studies on the overall results. We could assess the results' robustness and identify any influential studies by systematically removing one study at a time and recalculating the pooled effect estimate. This approach helps ensure that the conclusions drawn from the meta-analysis are reliable and not overly influenced by any single study.

**Publication bias:** We planned to assess publication bias using funnel plots and Egger's test. If there is evidence of publication bias, we would have conducted a trim-and-fill analysis to adjust for the bias. There should be at least 10 studies for publication bias studies in a meta-analysis by the funnel plot test; fewer studies might not give sufficient power to the test and may not detect the real asymmetry. 18 In this review, the number of studies on individual outcomes were less than 10. Therefore, publication bias detection was not performed.

## Results

### Study Selection and Characteristics

**Fig. 1:**
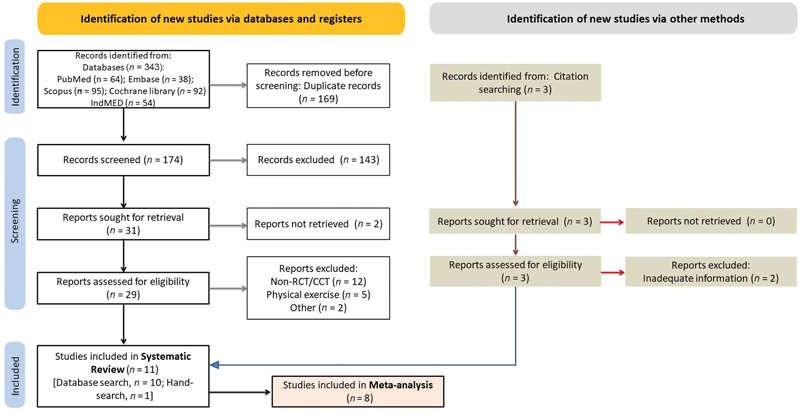
Study flow diagram. CCT, controlled clinical trial; non-RCT, nonrandomized controlled trial.

**Fig. 2:**
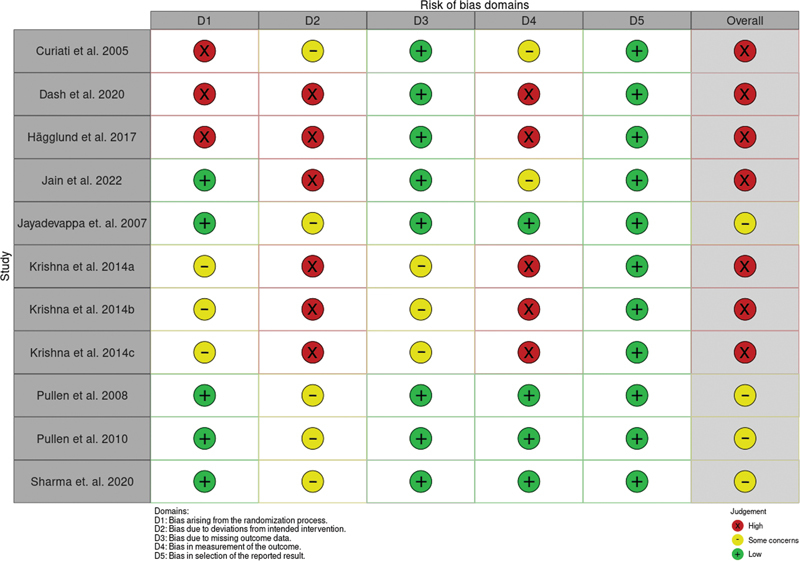
Risk of bias for individual studies.

**Fig. 3:**
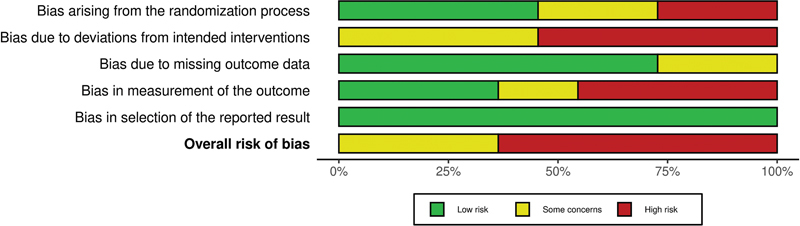
Risk-of-bias summary plot.

Our systematic review identified 343 articles from electronic databases and 3 articles from hand-searching of citations. After removing duplicates ( *n* = 169) and screening titles and abstracts, we excluded 143 articles that did not meet our inclusion criteria. We then reviewed the full texts of the remaining 34 articles and excluded 23 studies that were not retrievable or did not meet our eligibility criteria. Ultimately, we included 11 RCTs 4 7 8 9 19 20 21 22 23 24 25 in our systematic review ( Fig. 1 ), of which 8 studies 4 7 8 19 20 21 22 25 with 437 participants reported extractable outcomes and were included in the meta-analysis. Most of the studies ( *n* = 7, 63.64%) were evaluated as having a “high RoB,” 4 9 19 20 21 22 24 and the rest had projected “some concerns” 7 8 23 25 ( Figs. 2 , 3 ).

The 11 RCTs 4 7 8 9 19 20 21 22 23 24 25 involved 552 participants with CHF. The yoga interventions ranged from 8 to 24 weeks, with an average duration of 12 weeks reported in 70% of the studies. Five studies were conducted in India, 4 20 21 22 23 4 in the United States, 7 8 24 25 1 in Sweden, 9 and 1 in Brazil. 19 In most trials, yoga intervention was given in the context of standard care, while in two trials, yoga was compared with hydrotherapy 9 and guideline-based therapy. 20 The interventions included various compositions of yoga styles, such as meditation, yogic postures (asanas), breathing exercises (pranayama), and relaxation phases. Two studies 19 24 25 exclusively used meditation as an intervention. The control groups received either usual care or an alternative control intervention, such as hydrotherapy. No serious event was reported in any of the trials. We evaluated the effect of yoga on various outcomes in CHF patients through systematic review, with or without meta-analysis ( Table 2 ).

**Table 2:** Details of included studies

Study	Country	Duration	Participants	Groups	Inclusion	Existing therapy	Outcome measures	Intervention	Treatment	Control
Curiati et al 19	Brazil	12 wk	15	*M* = 8, CG = 7	NYHA I and II	Standard therapy	NE, MLwHFQ, peak VO _2_ and VE/VCO _2_ slope by cardiopulmonary exercise testing, LVEF, and LVDDi	Meditation	The meditation group was given a 30-min audiotape to listen to at home, twice per day, for 12 wk, plus a weekly meeting for guidance about the technique and group meditation	The control group had just a weekly meeting, which included talking about stress
Jain et al 20	India	12 wk	60	Yoga group (YG) = 30, CG = 30	NYHA I and II	Guideline-based therapy	MLwHFQ, CRP, NT-ProBNP, and LVEF	Asana, pranayama, and meditation	YG were given training (10-min asana + 30-min pranayama + 20-min meditation) in addition to guideline-based therapy, by a trained yoga instructor for 1 wk, after which patients were instructed to continue yoga daily for ∼60 min at home	Only guideline-based therapy
Krishna et al 21	India	12 wk	92	YG = 44, CG = 48	NYHA I and II	Standard therapy	MLwHFQ and 6-mWT	Pranayama, meditation, and relaxation	Each session lasted around 60 min. After 2 wk of participation in monitored sessions, YG patients practiced the same for 3 d under direct supervision and 3 d at their home	Only standard therapy
Krishna et al 22	India	12 wk	92	YG = 44, CG = 48	NYHA I and II	Standard therapy	LVEF, myocardial performance index (Tei index), and NT-ProBNP	Pranayama, meditation, and relaxation	Each session lasted around 60 min. After 2 wk of participation in monitored sessions, YG patients practiced the same for 3 d under direct supervision and 3 d at their home	Only standard therapy
Krishna et al 4	India	12 wk	92	YG = 44, CG = 48	NYHA I and II	Standard therapy	Heart rate, blood pressure, cardiac autonomic function (by short-term heart rate variability analysis), and myocardial oxygen consumption	Asana and pranayama	Yoga sessions lasted for 60 min and were conducted thrice per week, for a total of 36 supervised sessions over 12 wk	Only standard therapy
Pullen et al 8	United States	8 wk	19	YG = 9, CG = 10	NYHA I, II, and III	Standard therapy	Treadmill time, peak VO _2_ , weight, flexibility, MLwHFQ	Asana, pranayama, and meditation	Yoga sessions lasted for 70 min and twice per week, for a total of 16 supervised sessions over 8 wk. 10-minute warmup phase, a 40-min asanas, and 20-min relaxation phase including breathing exercises (pranayama) and meditation. After 2 wk of participation in monitored sessions, patients were instructed to perform at least 1 session at home for a minimum of 3 yoga sessions per week during the treatment period, with the help of video and handouts	Only standard therapy
Pullen et al 7	United States	8 wk	40	YG = 21, CG = 19	NYHA I, II, and III	Standard therapy	Treadmill time, flexibility, interleukin-6 (IL-6), CRP, peak VO _2_ , EC-SOD	Asana, pranayama, and relaxation	Yoga sessions for 60 min twice per week. Patients attended a total of 16 supervised sessions during an 8- to 10-wk period. Each session composed of 5-min warmup phase including pranayama, 40-min asanas, and 15-min relaxation phase. On completion of the first four classes, patients were given a handout of the 18 yoga postures taught during class to perform at least 1 session at home for a minimum of 3 yoga sessions per week during the treatment period	Only standard therapy
Sharma et al 23	India	12 wk	64	YG = 32, CG = 32	NYHA I and II	Standard therapy	LVEF, DASI, and metabolic equivalents (METs)	Asana, pranayama, and relaxation	60-min supervised and validated yoga module comprising asanas (physical postures), pranayama (breathing techniques), and relaxation techniques thrice a week for 12 wk along with the standard pharmacologic therapy prescribed for the condition	Only standard therapy
Aditee et al 24	United States	24 wk	25	*M* = 16, CG = 9	NYHA 2.2	Standard therapy	Primary: cumulative occurrence of AF; secondary: mortality, heart failure hospitalization, and ventricular arrhythmias	Vipassana meditation	Participants were given vipassana meditation technique, which involves focusing on the breath while being aware of any thoughts or sensations that arise and gently returning to the breath	Only standard therapy
Hägglund et al 9	Sweden	12 wk	30	Y = 18, HT = 12	NYHA I, II, and III	Hydrotherapy	EQ-5D, EQ-VAS, KCCQ, 6-mWT, sit-to-stand test, SBP, DBP, heart rate, saturation, hsCRP, and NT-ProBNP	Asana, pranayama, and relaxation/meditation	A 60-minute yoga session was conducted twice a week for 12 wk. A 10-min warmup phase including breathing exercises, a 40-min period of seated yoga postures and a 10-min relaxation/meditation phase	Hydrotherapy
Jayadevappa et al 25	United States	24 wk	23	TM = 13, HE = 10	NYHA II and III	Standard therapy	Primary: 6-mWT; secondary: MLwHFQ, SF-36, QWB-SA, and NT-ProBNP	Transcendental meditation	15–20 min twice daily while sitting comfortably with eyes closed	Health education

Abbreviations: 6-mWT, 6-minute walk test; CG, control group; CRP, C-reactive protein; DASI, Duke Activity Status Index; DBP, diastolic blood pressure; EC-SOD, extracellular superoxide dismutase; EQ-5D, EuroQol five descriptive dimensions; EQ-VAS, EuroQol Visual Analog Scale; hsCRP, high-sensitivity C-reactive protein; KCCQ, Kansas City Cardiomyopathy Questionnaire; LVDDi, left ventricular end-diastolic volume index; LVEF, left ventricular ejection fraction; M, meditation; MLwHFQ, Minnesota Living with Heart Failure Questionnaire; NE, nor epinephrine; NT-ProBNP, N-terminal prohormone of brain natriuretic peptide; NYHA, New York Heart Association; QWB-SA, the Quality of Well-Being Self-Administered; SBP, systolic blood pressure; SF-36, Short Form 36; VE/VCO2 slope, the minute ventilation/carbon dioxide production; peak VO _2_ , measure that combines cardiovascular and skeletal muscle oxidative function; Y, yoga.

### Left Ventricular Ejection Fraction

**Fig. 4:**
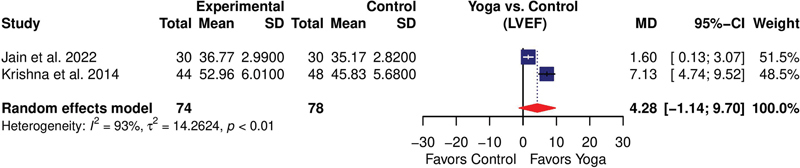
Forest plot showing the effect of yoga on the left ventricular ejection fraction (LVEF). CI, confidence interval; MD, mean deviation; SD, standard deviation.

Three studies 19 20 22 involving 167 participants evaluated the changes in LVEF after the yogic intervention. Krishna et al 22 showed a significant increase in LVEF in both the yoga and control groups. However, the increase in LVEF was significantly higher in the yoga group compared with the control group. Likewise, a study by Jain et al 20 showed a significant increase in LVEF in the yoga group, whereas the control group showed a nonsignificant increase. On the other hand, Curiati et al 19 showed no significant changes in LVEF in the yoga and control groups. All these studies used 12 weeks of intervention. The study by Curiati et al 19 was not included in the meta-analysis because of a wide baseline difference between groups. However, the pooled effect of the remaining studies did not show any significant improvement in LVEF, with high heterogeneity ( *I* ^2^ = 93%) and a mean difference of 4.28 (95%CI: –1.14 to 9.70) in random effect meta-analysis ( Fig. 4 ).

### NT-proBNP

**Fig. 5:**
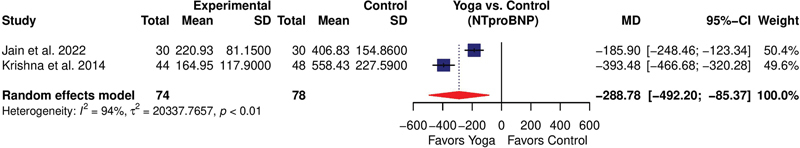
Forest plot showing the effect of yoga on serum NT-proBNP level. CI, confidence interval; MD, mean deviation; NT-proBNP level, N-terminal prohormone of brain natriuretic peptide; SD, standard deviation.

Three studies 20 22 25 reported changes in NT-proBNP after yoga intervention with a total of 175 participants. However, the two similar studies by Jain et al 20 and Krishna et al 22 were included in the meta-analysis. In these studies, NT-proBNP was reported in different units, and the values were converted to pmol/L before analysis. The pooled estimate showed a substantial reduction in NT-proBNP after the yogic intervention (MD = –288.78; 95%CI: –492.20 to –85.37; *I* ^2^ = 94%; Fig. 5 ).

### Quality of Life

In this systematic review, seven studies 7 8 9 19 20 21 25 evaluated the effect of yoga on the QoL of patients with CHF. Of these, six assessed the QoL using the Minnesota Living with Heart Failure Questionnaire (MLwHFQ). 7 8 19 20 21 25 In contrast, one study 25 additionally used 36-item Short Form (SF-36) survey and Quality of Well-Being Self-Administered (QWB-SA). Another study by Hägglund et al 9 used three different scales, that is, the Kansas City Cardiomyopathy Questionnaire (KCCQ), EuroQol five descriptive dimensions (EQ-5D), and EuroQol Visual Analog Scale (EQ-VAS).

Out of six studies that assessed QoL through MLWHFQ, three reported 19 20 21 significant improvement in the yoga intervention group. In contrast, the other three studies 7 8 25 reflected a nonsignificant improvement in the yoga group compared with the control group.

Among other outcomes of QoL, studies reported a significant increase in the social function domain of SF-36. 25 However, no improvement was observed in QWB-SA, 25 compared with standard care. The effect of yoga was found to be similar to hydrotherapy in changing EQ-5D, EQ-VAS, and disease-specific QoL measured using KCCQ. 9

**Fig. 6:**
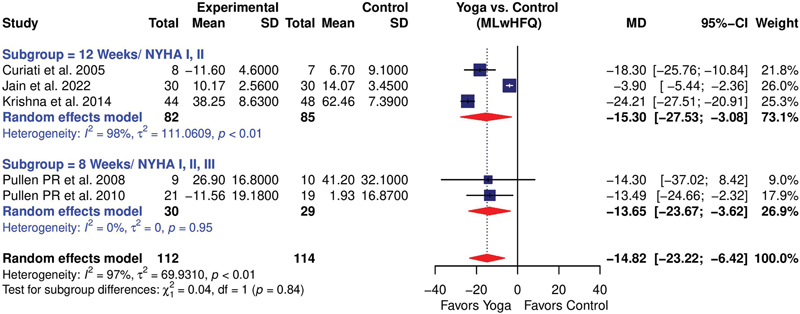
Forest plot showing the effect of yoga on MLwHFQ score (including all the studies). CI, confidence interval; MD, mean deviation; MLwHFQ, Minnesota Living with Heart Failure Questionnaire; SD, standard deviation.

**Fig. 7:**
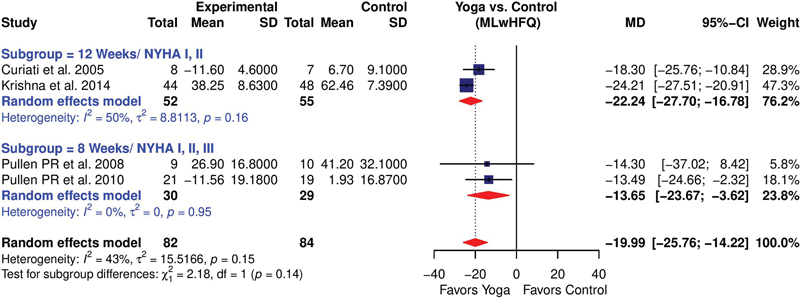
Forest plot showing the effect of yoga on MLwHFQ score (revised in sensitivity analysis). CI, confidence interval; MD, mean deviation; MLwHFQ, Minnesota Living with Heart Failure Questionnaire; SD, standard deviation.

Initially, six studies 7 8 19 20 21 25 that reported MLwHFQ were included in the meta-analysis. However, one study was excluded from the analysis 25 because of the vast dissimilarity in duration and nature of the intervention. Of these, three studies 19 20 21 used 12 weeks of intervention and included the NYHA I and II patients in the study, whereas the two remaining studies 7 8 used 8 weeks of intervention and included the NYHA I, II, and III patients. Both were analyzed as subgroups as well as cumulative. The first subgroup showed a high heterogeneity ( *I* ^2^ = 98%) and a mild treatment effect (MD = –15.30; 95%CI: –27.53 to –3.08). Excluding the study by Jain et al, 20 there was a significantly improved treatment effect (MD = –22.24; 95%CI: –27.70 to –16.78), with a substantial reduction in heterogeneity ( *I* ^2^ = 50%). The second subgroup showed a more robust and significant treatment effect (MD = –13.65; 95%CI: –23.67 to –3.62), with a very insignificant ( *I* ^2^ = 0%) heterogeneity. The overall effect in all the studies ( Fig. 6 ) was –14.86 (95%CI: –27.01 to –2.70; *I* ^2^ = 97%), and after excluding the study by Jain et al, 20 the pooled estimate improved to –19.99 (95%CI: –25.76 to –14.22; *I* ^2^ = 43%; Fig. 7 ).

### Peak VO _2_

**Fig. 8:**
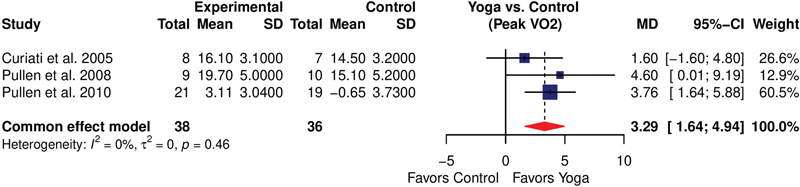
Forest plot showing the effect of yoga on changing peak VO _2_ . CI, confidence interval; MD, mean deviation; SD, standard deviation.

Peak VO _2_ was reported in three studies 7 8 19 ( *n* = 74) in the unit of mL/kg/min. The overall estimate following the intervention showed a significant improvement in peak VO _2_ (MD = 3.29 [95%CI: 1.64–4.94]). The heterogeneity among the studies was reported as very low, with an *I* ^2^ value of 0%, which suggests that the pooled result is robust and reliable ( Fig. 8 ).

### Six-Minute Walk Test

**Fig. 9:**
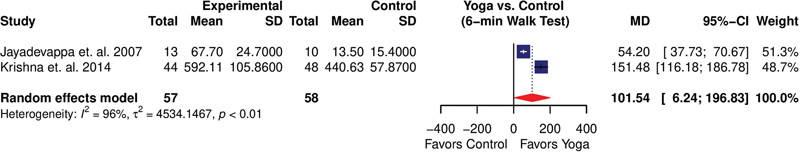
Forest plot showing the effect of yoga on 6-minute walk test. CI, confidence interval; MD, mean deviation; SD, standard deviation.

Two studies have measured the exercise capacity of HF patients using 6mWT in the unit of meters. The result of Krishna et al's study 21 showed a significant increase in 6mWT distance in the yoga group compared with the control group. Likewise, the study of Jayadevappa et al 25 showed a significant improvement in the 6mWT in the yoga group from baseline to 6 months after treatment compared with the control (health education) group. The meta-analysis showed a significant effect of the intervention in improving the 6mWT (MD = 101.54; 95%CI: 6.24–196.83, *I* ^2^ = 96%; Fig. 9 ).

### Other Outcomes

One study 22 evaluated the effect of 12 weeks of yoga on the Myocardial Performance Index (Tei index). The Tei index is a straightforward, reliable, and independent indicator of heart rate and blood pressure of total cardiac dysfunction in patients with mild to moderate HF. It is calculated as the sum of isovolumic contraction and relaxation time divided by the ejection time. The study showed a significant reduction in the Tei index in the yoga group compared with the control group.

In some studies, it has been observed that yoga led to significant improvement in heart rate, 4 increase in high-frequency spectrum and decrease in low-frequency spectrum of HRV, measured through Kubios HRV Version 2.0 software for HRV (Bio-Signal Analysis Group, Finland), 4 reduction in systolic blood pressure (SBP) and diastolic blood pressure (DBP), 4 improvement in rate pressure product (RPP), 4 reduction in hsCRP 7 8 20 and interleukin-6 (IL-6), 7 8 increase in extracellular superoxide dismutase (EC-SOD) activity, 7 8 increase in treadmill time, 7 8 decrease in the minute ventilation/carbon dioxide production (VE/VCO _2_ ) slope, 19 and reduction in the norepinephrine level. 19

Yoga did not affect some outcomes like cortisol levels 25 and left ventricular end-diastolic volume index (LVDDi). 19 A study reported 9 that the effect of yoga may be similar to hydrotherapy in various outcomes like HRQoL, peripheral oxygen saturation, heart rate, SBP and DBP, high-sensitivity C-reactive protein (hs-CRP), and NT-pro BNP.

Aditee et al 24 assessed the effect of yoga on clinical outcomes in patients with implantable defibrillators for HF. The study showed a reduction in antiarrhythmic use, cumulative atrial fibrillation (AF), persistent AF, sustained VA, ablation for HF, HF hospitalization, and an increase of survival in the yoga group compared with the control group. 24

## Discussion

### Principal Findings

The systematic review including 11 randomized trials with 552 participants aimed to investigate the effects of yoga as an adjunct therapy for patients with CHF. The principal findings of the review indicate that yoga interventions may have beneficial effects on various outcomes such as QoL, peak VO _2_ , exercise capacity, endurance, and a few cardiac biomarkers such as NTproBNP. Subgroup analysis suggested that the effects of yoga on QoL were more pronounced in patients with the NYHA class I and II CHF patients and in those who practiced yoga for longer durations. The review found no significant effect of yoga on LVEF in patients with CHF. However, the evidence is limited, and the quality of the studies included is generally low.

### Strengths and Limitations

One of the strengths of this review is that it is based on a comprehensive search of several databases, which included exclusively RCTs. Additionally, the review followed the PRISMA guidelines for conducting and reporting systematic reviews, which enhances its reliability and validity. However, the review has some limitations that need to be considered. One of the main limitations is the small number of studies included in the review, which limits the generalizability of the findings. Moreover, the quality of the studies included in the review is generally low, with several studies having a high RoB. In the meta-analysis, very few studies could be included considering the similarity in individual outcomes. The involved heterogeneity was another concern while interpreting the study results. Only the QoL could be explained a little, using subgroup and sensitivity analyses. The quality of reporting was another issue found in most of the trials, which carries a negative dimension while interpreting such reports.

### Comparison with Previous Similar Studies

Our findings are generally consistent with a previous systematic review 16 that reported that yoga might improve peak VO _2_ and HRQoL in patients with CHF. However, the previous review has included fewer studies ( *n* = 2), and significant time has elapsed since the review. Our review adds to the literature by including a more comprehensive search of the literature, including a broader range of studies, and evaluating the quality of the studies extensively using validated tools. In contrast to the previous one, this review evaluated the effect of yoga on a broader range of clinical outcomes in CHF patients.

### Future Direction and Recommendation

The findings of this review suggest that yoga may have potential benefits as an adjunct therapy for patients with CHF. However, evidence is limited, and the quality of the studies is generally low. Future studies should focus on conducting high-quality RCTs with larger sample sizes to provide more robust evidence on the effects of yoga in patients with CHF. Moreover, future studies should also aim to evaluate the long-term effects of yoga on various outcomes in this population. Given the heterogeneity of yoga interventions used in the studies included in this review, future studies should aim to standardize the interventions to allow for better comparison across studies. In addition, the quality of comprehensive reporting and adverse events must be strengthened in future yoga trials.

## Conclusion

This systematic review suggests that yoga may have some benefits as an adjunct therapy for patients with CHF. The review found that yoga interventions may improve QoL, peak VO _2_ , exercise capacity, endurance, and cardiac biomarkers. However, the evidence is limited and the quality of the studies included in the review is generally low, which limits the generalizability of the findings. Therefore, future studies should focus on conducting high-quality RCTs with larger sample sizes to provide more robust evidence on the effects of yoga in patients with CHF. Additionally, future studies should aim to standardize the interventions to allow for better comparison across studies and evaluate the long-term effects of yoga on various outcomes in this population. Overall, the findings of this review provide some promising initial evidence for the potential use of yoga as an adjunct therapy for patients with CHF, but further research is needed to evaluate its efficacy and safety beyond any doubt.
